# Supplementary material for: Ribitol in Solution Is an Equilibrium of Asymmetric Conformations
Source: Molecules. 2021 Sep 8;26(18):5471. doi: 10.3390/molecules26185471 (PMC8468352; doi:10.3390/molecules26185471)
Supplement: Supplementary file 1 [file molecules-26-05471-s001.zip › molecules-1354125-supplementary.pdf]

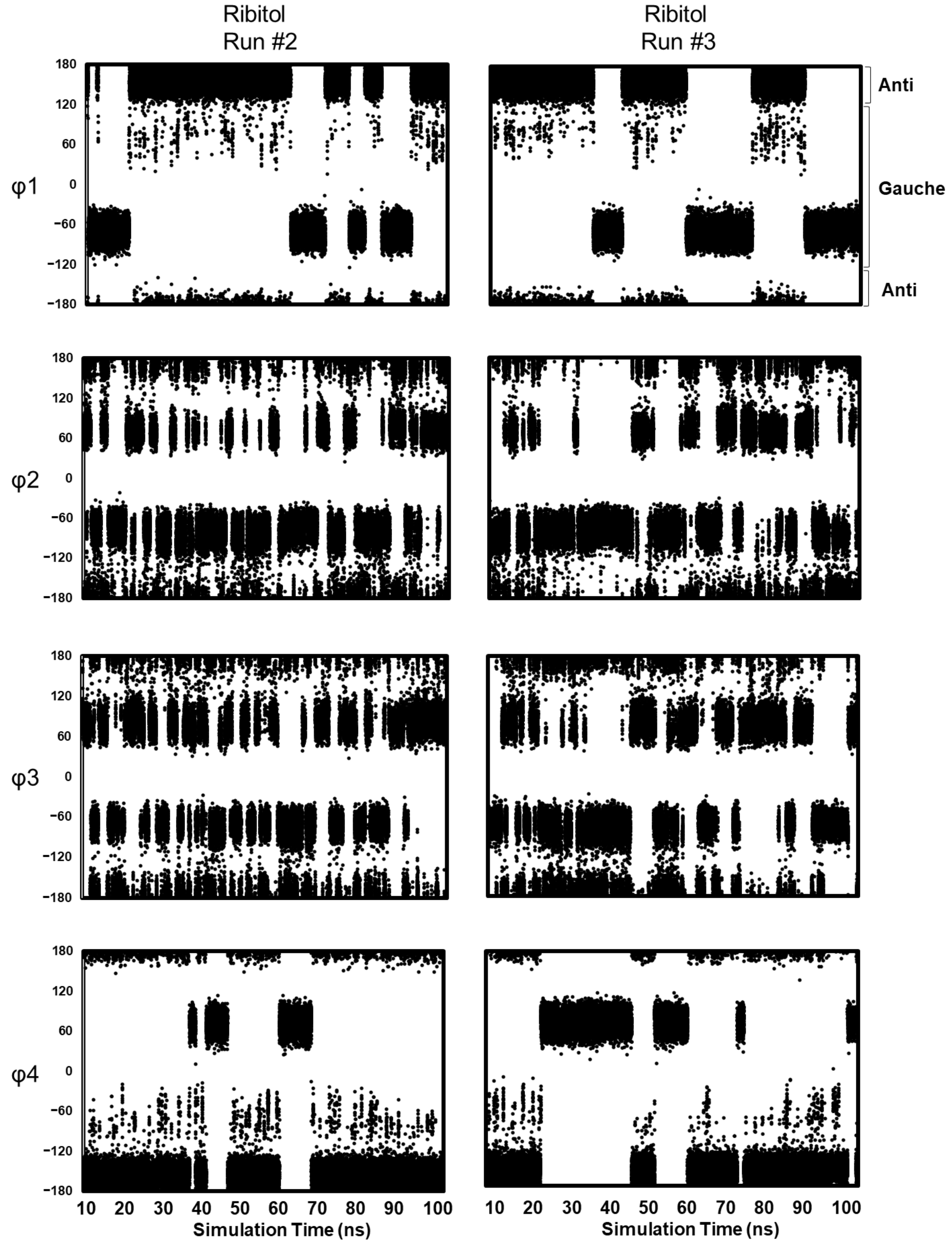

Supplementary Figure S1. Dihedral angle distribution of ribitol in MD simulation Run #2 (left) and Run #3 (right). The results of each dihedral angle are shown after 10 ns. The dihedral angles are defined as φ1 (O1-C1-C2-C3), φ2 (C1-C2-C3-C4), φ3 (C2-C3-C4-C5), and φ4 (C3-C4-C5-O5).

PDB ID	Atom	X	Y	Z
5IAI	C1	56.7	8.2	50.0
	O1	55.7	9.2	50.3
	C2	57.0	7.5	51.3
	O2	57.7	8.4	52.2
	C3	58.0	6.3	51.0
	O3	57.3	5.3	50.3
	C4	58.7	5.7	52.2
	O4	59.6	4.7	51.8
	C5	57.7	5.2	53.2
	O5	58.3	4.5	54.3
4Q0S	C1	8.6	7.1	32.8
	O1	9.0	7.9	33.9
	C2	8.8	7.8	31.5
	O2	8.0	9.0	31.6
	C3	8.2	6.9	30.4
	O3	6.9	6.6	30.6
	C4	9.0	5.6	30.2
	O4	10.4	5.9	30.3
	C5	8.6	5.0	28.9
	O5	9.4	3.8	28.6
4F2D	C1	99.7	-4.4	18.1
	O1	100.3	-3.3	18.8
	C2	99.1	-5.4	19.1
	O2	99.9	-5.4	20.3
	C3	99.2	-6.8	18.6
	O3	98.5	-6.9	17.3
	C4	98.5	-7.8	19.6
	O4	98.9	-9.1	19.3
	C5	97.0	-7.7	19.5
	O5	96.4	-7.9	20.8

Supplementary Table S1. Initial coordinates of ribitol for MD simulations. Three crystal structures of ribitol are used for three independent MD simulations Run #1~#3.

	Ribitol (Run #1)			Ribitol (Run #2)			Ribitol (Run #3)			1,3,5-pentanol		
	180°	60°	−60°	180°	60°	−60°	180°	60°	−60°	180°	60°	−60°
φ1	78.6	1.6	19.8	65.7	1.2	33.1	58.2	1.1	40.8	56.7	19.1	24.3
φ2	14.5	35.2	50.3	17.8	27.8	54.4	18.2	26.0	55.8	67.9	4.3	27.8
φ3	14.9	47.0	38.1	16.2	46.9	36.9	18.1	40.5	41.4	69.2	26.5	4.3
φ4	66.9	31.3	1.9	81.5	16.5	2.1	61.5	37.3	1.3	57.2	20.8	22.0

Supplementary Table S2. The population (%) of dihedral angles in MD simulations of ribitol (Run #1~#3) and 1,3,5-pentanol. −120° to −180° and 120° to 180° is categorized as 180°, 0° to 120° is categorized as 60°, −120° to 0° is categorized as −60°.
